# Zika virus infection of retinal cells and the developing mouse eye induces host responses that contrasts to the brain and dengue virus infection

**DOI:** 10.1007/s13365-023-01123-5

**Published:** 2023-04-06

**Authors:** E. Cowell, L. P. Kris, G. Bracho-Granado, H. Jaber, J. R. Smith, J. M. Carr

**Affiliations:** 1 Microbiology and Infectious Diseases, College of Medicine and Public Health, Flinders University, Room 5D-316, Flinders Medical Centre, Flinders Drive, Bedford Park, Adelaide, South Australia 5042 Australia; 2 Eye and Vision Health, College of Medicine and Public Health, Flinders University, Bedford Park, Adelaide, South Australia 5042 Australia

**Keywords:** Dengue virus, Zika virus, Eye infection, Retina, Development, Inflammation, Nanostring

## Abstract

Zika virus (ZIKV) infection causes ocular and neurological pathologies with ZIKV-induction of developmental abnormalities following in utero infection a major concern. The study here has compared ZIKV and the related dengue virus (DENV) infection in the eye and brain. In vitro, both ZIKV and DENV could infect cell lines representing the retinal pigmented epithelium, endothelial cells, and Mueller cells, with distinct innate responses in each cell type. In a 1-day old mouse challenge model, both ZIKV and DENV infected the brain and eye by day 6 post-infection (pi). ZIKV was present at comparable levels in both tissues, with RNA increasing with time post-infection. DENV infected the brain, but RNA was detected in the eye of less than half of the mice challenged. NanoString analysis demonstrated comparable host responses in the brain for both viruses, including induction of mRNA for myosin light chain-2 (Mly2), and numerous antiviral and inflammatory genes. Notably, mRNA for multiple complement proteins were induced, but C2 and C4a were uniquely induced by ZIKV but not DENV. Consistent with the viral infection in the eye, DENV induced few responses while ZIKV induced substantial inflammatory and antiviral responses. Compared to the brain, ZIKV in the eye did not induce mRNAs such as C3, downregulated Retnla, and upregulated CSF-1. Morphologically, the ZIKV-infected retina demonstrated reduced formation of specific retinal layers. Thus, although ZIKV and DENV can both infect the eye and brain, there are distinct differences in host cell and tissue inflammatory responses that may be relevant to ZIKV replication and disease.

**Supplementary Information:**

The online version contains supplementary material available at 10.1007/s13365-023-01123-5.

## Introduction

Zika virus (ZIKV) and dengue virus (DENV) are closely related mosquito-transmitted, flaviviruses, and both are associated with a febrile illness (Guzman and Harris 2015; Musso and Gubler 2016; White et al. 2016). DENV infection can result in serious outcomes—dengue with warning signs and severe dengue, associated with a vascular leak syndrome which is seen in both adults and children. In contrast, ZIKV infection in adults is largely asymptomatic, with generalised rash, headache, and fever (Halani et al. 2021). ZIKV, however has been recognised as a pathogen with damaging effects on development of the brain and the retina, with a diverse clinical presentation termed congenital zika syndrome (CZS) (Agrawal et al. 2018; de Paula Freitas et al. 2017; Musso and Gubler 2016; Musso et al. 2019).

One commonality between the diseases caused by ZIKV and DENV is eye involvement, although the specifics of this are quite different, with the clinical characteristics of these infections reviewed previously (Merle et al. 2018; Oliver et al. 2019). ZIKV is commonly associated with a conjunctivitis in adults (de Paula Freitas et al. 2017; Halani et al. 2021) or anterior uveitis, both of which are infections and inflammation in the anterior eye. In contrast, DENV has been well described to cause a retinopathy, including retinal vascular disease in the posterior eye (Li et al. 2017; Lim et al. 2004; Teoh et al. 2006). Our laboratory has demonstrated that DENV can infect cell types from the retina (Carr et al. 2017), and DENV can infect the eye in animal models of adult disease (Norbury et al. 2020). ZIKV has been demonstrated in the eye in models of in utero or post-natal mouse development (Li et al. 2021; Zhao et al. 2017), infection in adult mice when the IFN-response is lacking (Garcia et al. 2020; Miner et al. 2016; Singh et al. 2019), and CZS can be studied in a number of animal models in the laboratory (Caine et al. 2018; Narasimhan et al. 2020). In relation to infection of the brain, DENV can cause central nervous system disease, such as encephalitis in the adult, although rarely, (Li et al. 2017) but ZIKV is a well-recognised cause of microcephaly following infection in utero (de Paula Freitas et al. 2017; Musso and Gubler 2016; Musso et al. 2019). In the laboratory, DENV can infect the brain of adult mice and replicate in microglia, neurons, oligodendrocytes, and endothelial cells (Amorim et al. 2019; Velandia-Romero et al. 2012) and can move from the brain to the eye (Norbury et al. 2020), while ZIKV can infect the developing mouse brain (Noguchi et al. 2020) and isolated or organoid cultured neuronal progenitor cells (NPC) (Qian et al. 2017).

Here, we have extended these studies to define ZIKV infection and inflammatory responses in retinal cells in vitro and the developing neonatal mouse eye and brain early in infection and prior to the onset of neurological deficits. Further, viral infection and host responses are compared and contrasted to a laboratory strain of DENV. Results show that both viruses can infect eye cells in vitro and the brain and eye in vivo, with ZIKV replication comparable in both tissues during development. Both conserved and distinct virus, cell-line and tissue specific responses are observed reflecting differences in host antiviral responses, pro-inflammatory and developmental pathways that may be important for the specific ZIKV-induced changes in neuronal and retinal developmental dysfunction in the brain and eye.

## Materials and methods

### Cell lines

Generation and characterisation of the human retinal endothelial cells (HREC) line have been described previously (Bharadwaj et al. 2013). The HREC line was cultured in MCDB-131 medium (Sigma-Aldrich) with 5% FBS and endothelial growth factors (EGM-2 SingleQuots supplement, omitting FBS, hydrocortisone, and gentamicin; Clonetics-Lonza, (Walkersville, MD). The ARPE-19 cell line (American Type Culture Collection, Manassas, VA) was cultured in DMEM:F12 supplemented with 5% FBS. The MIO-M1 (Moorfields Eye Hospital/University College London Institute of Ophthalmology—Muller 1) retinal Muller-glial cell line, provided by Drs G. Astrid Limb and Peng T. Khaw, was cultured in DMEM supplemented with 10% FBS. All cells were grown in a humidified incubator with 5% CO_2_ in air and at 37 °C.

### Virus and infection

Mon601, a modified laboratory clone of the DENV-2 New Guinea C strain (Gualano et al. 1998), was used for in vitro DENV-infections. Mon601 stocks were produced from in vitro transcribed RNA that was transfected into BHK-21 cells and amplified in C6/36 cells. ZIKV infections utilised the ZIKV strain, PRVABC59, that was amplified in C6/36 cells. Cell culture supernatants containing virus were harvested, clarified, filtered, and stored at − 80 °C until use. The titer of infectious virus was determined by plaque assay using Vero cells and quantitated as plaque forming unit (pfu) per ml. For DENV and ZIKV infection in vitro, cells were seeded in 6-well culture plates at 3 × 10^5^ per well and mock or DENV-infected the following day for 90 min in serum-free media at a multiplicity of infection (MOI) of 1. Inoculum was removed, cells are washed with phosphate-buffered saline (PBS), complete medium are added, and cells are incubated until the required time point post infection (pi), where cells were lysed in TRIzol reagent for RNA extraction and RT-PCR.

### Mouse developmental model of infection

One-day old Balb/c pups were injected into the body just above the milk spot with 5000 PFU of DENV or ZIKV in a total volume of 10 μl. Mice were visually monitored daily for colour and movement. At the designated time post infection, mice were weighed and humanely killed by decapitation, eyes and brain harvested into TRizol, and stored for RNA extraction or fixed in 10% buffered formalin for sectioning and histological analysis.

### RNA extraction and RT-PCR

Total RNA was extracted from cells and mouse tissues using TRIzol, DNase I treated, and 0.5 μg RNA was reverse transcribed with 30 µM random hexamers and M-MuLV reverse transcriptase. cDNA template was subjected to real-time qRT-PCR using iTaq SYBER green in a Rotor-gene iQ (Qiagen), using primers listed in Table 1. All PCRs were performed under the following conditions: one cycle of 95 °C for 5 min; 40 cycles of 95 °C for 15 s, 58 °C for 30 s, and 72 °C for 30 s; and one cycle of 72 °C for 5 min. All PCR reactions included high and low copy number comparative controls. Results were normalised against the reference housekeeping genes: cyclophilin (for human PCR) or glyceraldehyde-3-phosphate dehydrogenase (GAPDH, for mouse PCR). The relative RNA level was determined by ΔCt method as described previously (Schmittgen and Livak 2008).

**Table 1:** Summary of primers utilised for qRT-PCR

Name	Species	Primer sequence	Accession no
DENV-2		*Forward* GCAGATCTCTGATGAATAACCAAC *Reverse* TTGTCAGCTGTTGTACAGTCG	D00346.1
Capsid region for Mon601			
ZIKVENV		Forward GCTGGDGCRGACACHGGRACT Reverse RTCYACYGCCATYTGGRCTG	
Envelope region for ZIKV PRVABC59			
Cyclophilin	Human	Forward GGCAAATGCTGGACCCAACACAAA Reverse CTAGGCATGGGAGGGAACAAGGAA	NM_021130.5
IFN-α	Human	Forward CAAGCCCAGAAGTATCTGCAATATC Reverse ACCAGGACCATCAGTAAAGCAAA	NM_024013.3
IFN-β	Human	Forward AGGTAGTAGGCGACACTGTTCGT Reverse AGAAGCACAACAGGAGAGCAATT	NM_002176.4
Viperin	Human	Forward GTGAGCAATGGAAGCCTGATC Reverse GCTGTCACAGGAGATAGCGAGAA	NM_080657.5
IL-6	Human	Forward AGACAGCCACTCACCTCTTCAG Reverse TTCTGCCAGTGCCTCTTTGCTG	NM_000600.5
TNF-α	Human	Forward CCCCAGGGACCTCTCTCTAATC Reverse GGTTTGCTACAACATGGGCTACA	NM_000594.4
CXCL10	Human	Forward TCCACGTGTTGAGATCATTGC Reverse TCTTGATGGCCTTCGATTCTG	NM_001565.4
GAPDH	Mouse	Forward GACGGCCGCATCTTCTTGTGC Reverse TGCCACTGCAAATGGCAGCC	NM_008084.3
Complement C2	Mouse	Forward CTCATCCGAGTTTACTCCAT Reverse TGTTCTGTTCGATGCTCAGG	NM_013484.2
Complement C3	Mouse	Forward CGCAACGAACAGGTGGAGATCA Reverse CTGGAAGTAGCGATTCTTGGCG	NM_009778.3

### NanoString nCounter gene expression assay

Immune gene expression analysis of total RNA (*n* = 4 mock, *n* = 3 DENV; *n* = 4 ZIKV) was performed using the NanoString™ GX nCounter^®^ Mouse Immunology Panel (NanoString Technologies, Seattle, WA, USA) on the Gen 2 nCounter^®^ FLEX Analysis System at the Systems Biology and Data Science Facility (Griffith University, Gold Coast, Australia) according to the manufacturer’s instructions. The panel contains 549 genes that includes six positive controls, eight negative controls, and five housekeepers for normalisation to account for platform and technical variability. Immune gene expression (nCounter) data was analysed using a combination of the Advanced Analysis Module in the nSolver™ Analysis Software version 4.0 from NanoString Technologies (NanoString Technologies, WA, USA) and customised scripts in the R statistical computing environment. Data analysis included quality control (QC), normalisation, and between group comparisons for differential gene expression (DGE) and pathway analysis.

### Histological analysis

Mouse eyes were enucleated and fixed in 10% (v/v) buffered formalin. Tissue was embedded in paraffin blocks, and 5-µm sections were cut and mounted onto glass slides. Sections were stained with haematoxylin and eosin (H&E) and examined under brightfield microscopy (VS200 Slide Scanner, Olympus) with the VS200 DESKTOP software used for image analysis and measurement of defined layers within the retina (in µm).

### Statistical analysis

Results were expressed as the mean ± standard deviation (SD), and statistical analyses were performed using a two-tailed unpaired Student’s *t*-test, one-way or two-way analysis of variance (ANOVA), or the non-parametric Kruskal–Wallis test for data that contained low values of *n*. Statistical analysis was performed using GraphPad Prism (GraphPad, La Jolla, CA, USA). Differences were considered statistically significant if *p* < 0.05.

### Ethics and biosafety

Mice were kept on a 12-h cycle of light and darkness with ad libitum access to food and water and in a pathogen-free environment. Procedures were performed in accordance with Flinders University Animal Welfare Committee approval number 939/17 and Institutional Biosafety Committee approval NLRD 2013–24.

## Results

### ZIKV and DENV infect eye cell lines and induce different cell response profiles

**Fig. 1:**
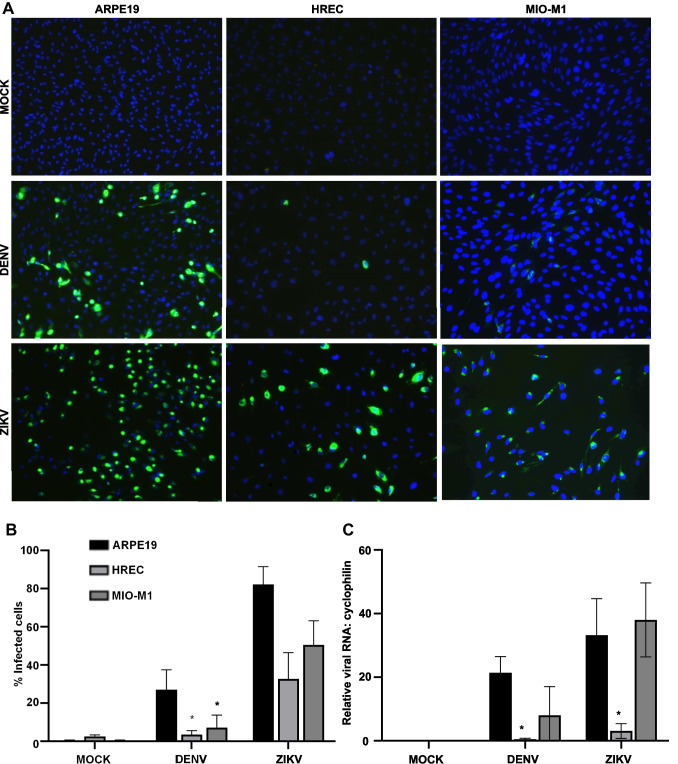
APRE-19, HREC, and MIO-M1 cells are susceptible to ZIKV and DENV infection. Cells were mock-, ZIKV-, or DENV-infected at MOI = 1 and at 48 hpi, fixed, and stained for viral antigen (4G2 and goat anti-mouse IgG-AlexaFluor488) and cell nuclei (Hoescht). Epifluorescent images were collected (Olympus fluorescence microscope, × 20 magnification), and at least 20 images each containing over 100 cells per condition were analysed with CellProfiler 2.2.0. **A** Representative epifluorescent images from each cell type. **B** Frequency of antigen positive cells. **C** At 48 h, pi cells were lysed, viral RNA quantitated by RT-PCR, and normalised against cyclophilin. Results represent mean ± SD from *n* = 3 replicates from a representative experiment (*n* = 2). Data were analysed by 2-way ANOVA with Tukey’s multiple comparison test. *p < 0.05

The ability of DENV and ZIKV to replicate in cell lines representing different cell types in the retina, retinal pigment epithelial cells (ARPE-19), retinal endothelial cells (HREC), and Mueller cells (MIO-M1), was defined. Cells were stained for viral antigens at 48 hpi, demonstrating clear DENV and ZIKV-infected cells in all cell types (Fig. 1A). ZIKV-infection resulted in APRE19 > HREC > MIO-M1 in terms of the number of antigen positive cells, while DENV demonstrated a greater number of infected ARPE-19 but comparable low numbers of infected HREC and MIO-M1 cells (Fig. 1B). RNA was extracted and viral RNA and host mRNA’s quantitated by RT-PCR. Consistent with the immunofluorescent antigen staining, ARPE-19 cells contained higher levels of either DENV or ZIKV RNA than HREC (Fig. 1C) (*p* = 0.0029 and *p* =  < 0.0001, respectively). Surprisingly, both DENV and ZIKV-infected MIO-M1 had a lower percentage of infected cells, but viral RNA levels in MIO-M1 cells were high and comparable to those in ARPE-19 (Fig. 1C).

**Fig. 2:**
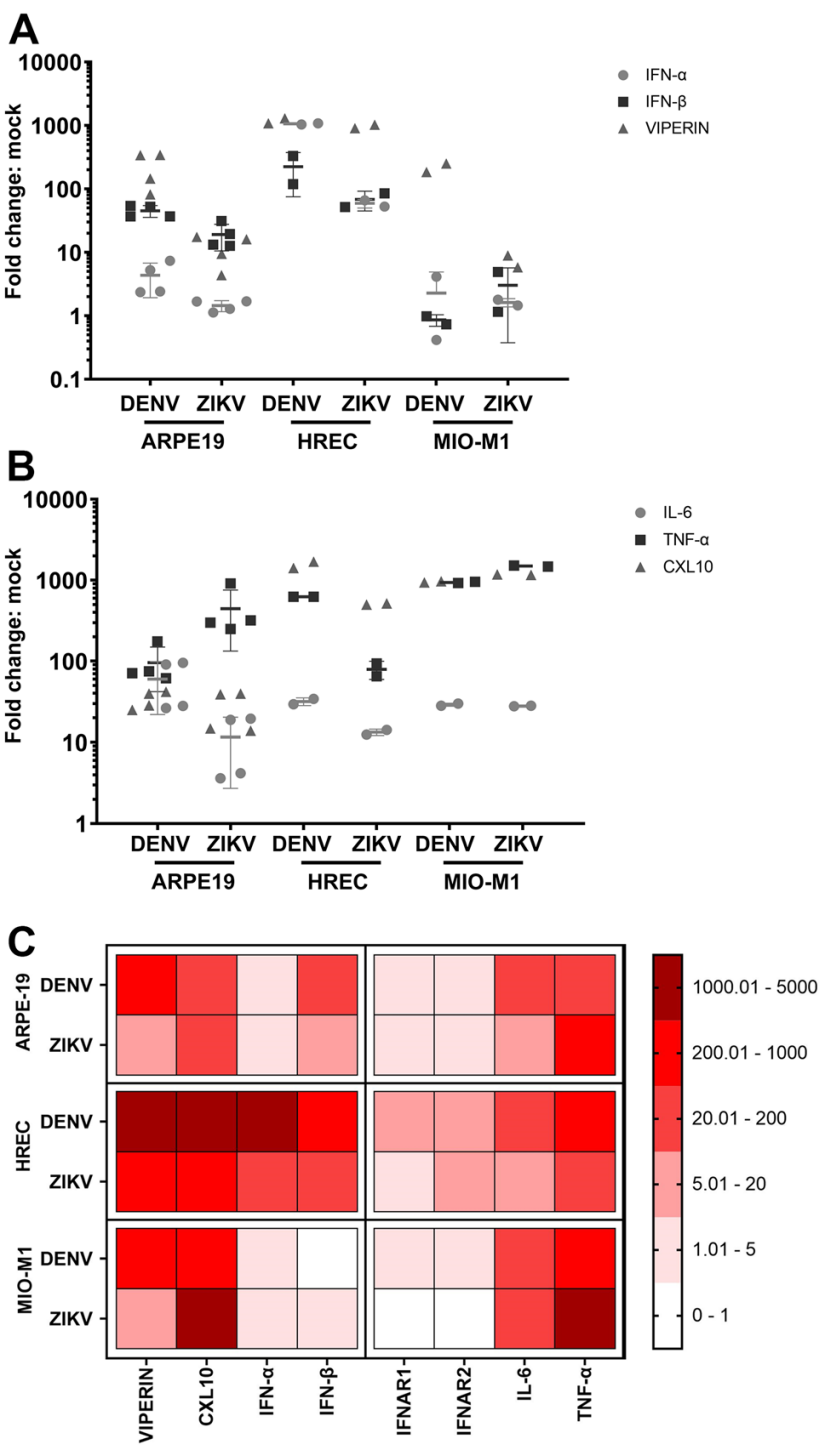
Relative expression of host cell factors in ZIKV- or DENV-infected cells. Total RNA from mock-, ZIKV-, or DENV-infected cells was extracted at 48 hpi and subjected to qRT-PCR. Data were normalised to a house-keeping gene (cyclophilin) and the ratio of each gene relative to basal mRNA levels in mock-infected cells. **A** Type I IFN and antiviral response, IFN-α and β, and viperin. **B** Inflammatory response, CXCL10, TNF-α, and IL-6. **C** Heat map representation of data. Results represent mean ± SD from *n* = 3 replicates from *n* = 2 independent experiments

The three different retinal cell lines were next analysed for components of the type I IFN system, antiviral, and inflammatory responses by qRT-PCR. Distinct virus and cell type profiles of induction for type I IFN (IFN-α/β) were seen with greatest responses induced by DENV in HREC (Fig. 2A), despite a lower level of infection compared to ZIKV (Fig. 1). Viperin was strongly induced by both DENV and ZIKV in HREC but only by DENV in ARPE-19 and MIO-M1 (Fig. 2A). Inflammatory markers, CXCL10, IL6, and TNF-α were induced in all cell lines and by both viruses, with generally lower levels of IL6 in HREC and MIO-M1 cells infected with either DENV or ZIKV (Fig. 2B). These results are summarised as a heat map (Fig. 2C).

### ZIKV and DENV infection differentially infects the eye and brain of the developing mouse

**Fig. 3:**
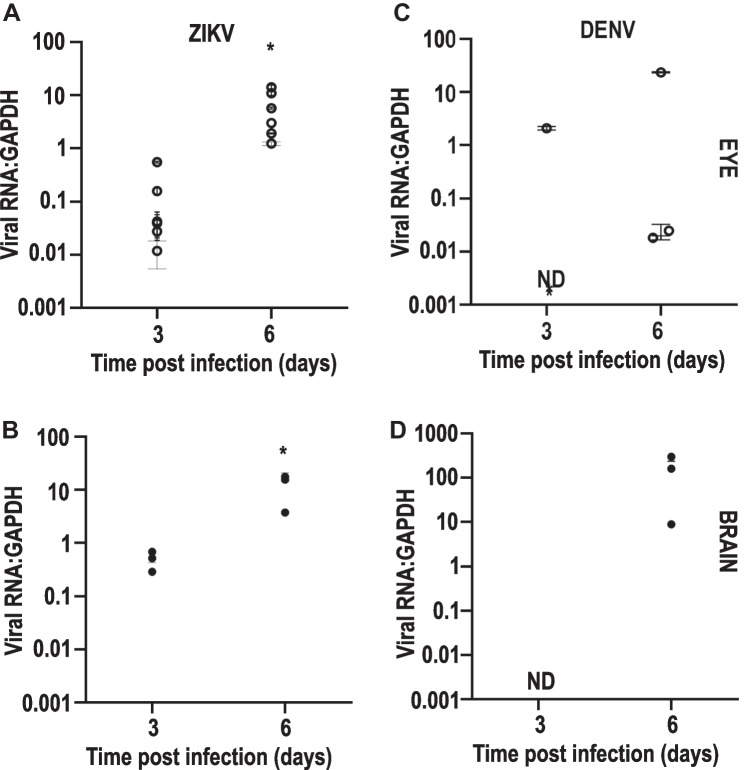
Viral RNA in eyes and brain of newborn mice infected with DENV or ZIKV. Newborn Balb/c were infected by injection of 5000 PFU of either DENV or ZIKV at day 1 post-natal and total RNA extracted from eye (*n* = 6) and brain (*n* = 3) at 3 or 6 days pi and subjected to qRT-PCR with results normalised to the housekeeping gene, GAPDH. **A** ZIKV RNA in the brain. **B** ZIKV RNA in the eye. **C** DENV RNA in the brain. **D** DENV RNA in the eye. Horizontal lines and errors bars represent the mean and standard deviation of normalised values respectively. Dots represent average data from individual animals. Data were analysed by Student’s *t*-test or one-way ANOVA and Tukey’s multiple comparisons test, * = *p* < 0.05

The above in vitro studies demonstrate that both ZIKV and DENV can target multiple cell types of the retina in different ways. Next, infection of the eye and brain was assessed in a newborn mouse model of systemic ZIKV and DENV challenge during development. Virus was injected into the body of 1-day old immunocompetent Balb/c mouse pups and viral RNA in the eye and brain quantitated by RT-qPCR. In the brain, low levels of ZIKV RNA were detected at day 3 pi, increasing at day 6 pi (Fig. 3A, p = 0.002). For DENV, at day 3 pi, no viral RNA was detected in the brain, but all mouse brains were infected by day 6 pi (Fig. 3B). Similarly, in the eye at day 3 pi, low levels of ZIKV RNA were detected in all eyes, with a significant increase at day 6 pi (Fig. 3C, p = 0.005). DENV RNA was variably detected in the eye, in one eye of one animal at day 3pi and in one eye in each of 3 animals at day 6pi (Fig. 3D).

**Fig. 4:**
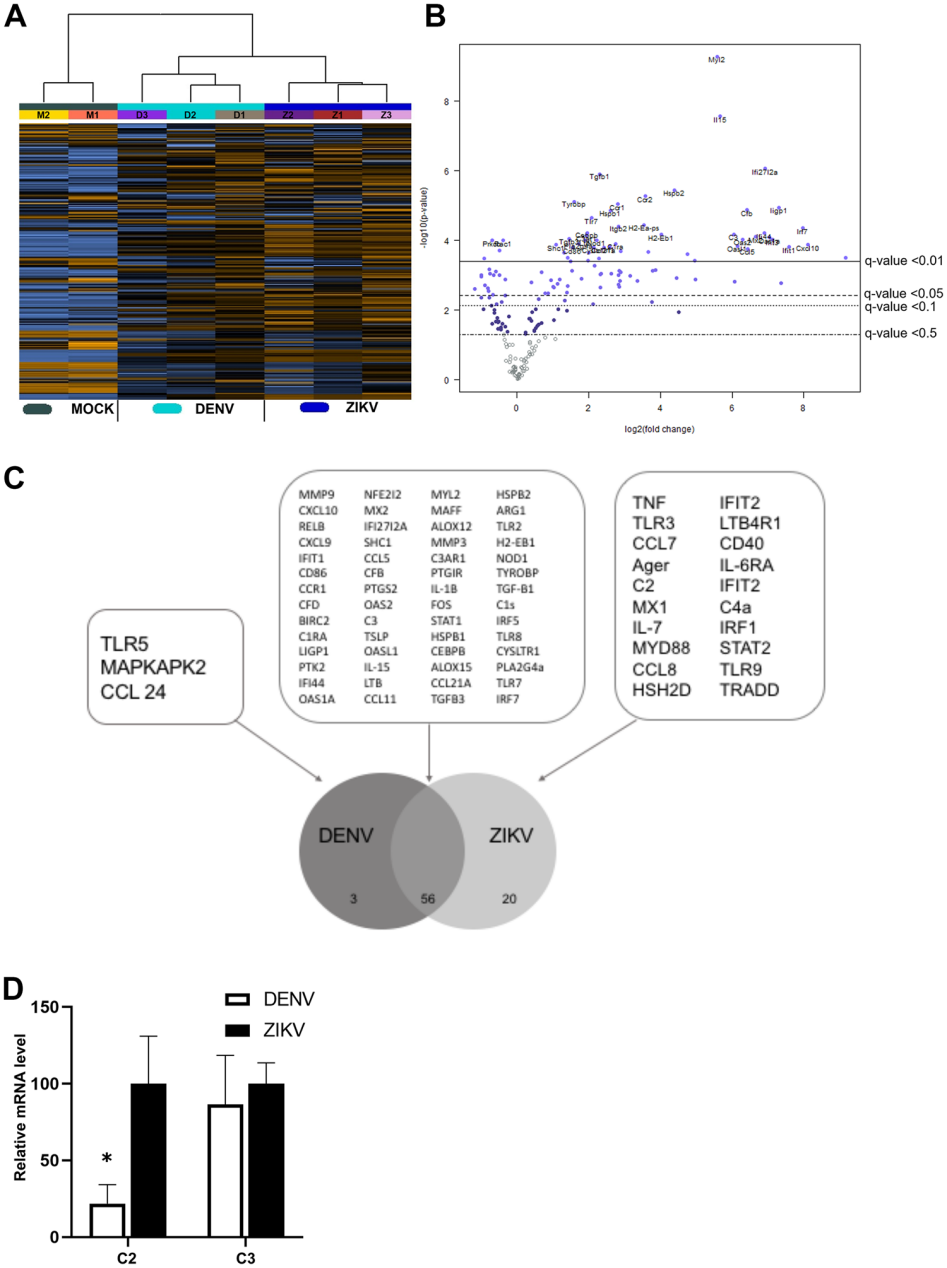
ZIKV and DENV induce comparable responses in the developing brain. Newborn Balb/c were mock (*n* = 2) or virally infected by injection of 5000 PFU of either DENV (*n* = 3) or ZIKV (*n* = 3), total RNA extracted from the brain at six days pi, and subjected to NanoString nCounter analysis for a panel of mouse inflammatory-related genes.** A** Hierarchical clustering and heat map. **B** Volcano plots of mock vs ZIKV. **C** Venn diagram of common and unique mRNA’s significantly upregulated by ZIKV or DENV infection of the developing brain. Individual gene names can be interrogated from Table 2. **D** C3 and C2 mRNA were quantitated by RT-PCR and normalised against GAPDH. Results represent mean ± SD from *n* = 3 replicates. Data were analysed by unpaired *t*-test, *p < 0.05

To analyse the inflammatory response, RNA from the brain and eye at day 6 pi was subjected to NanoString analysis for a panel of mouse inflammatory-associated genes. Hierarchical clustering of brain responses demonstrate induction of mRNA changes in both ZIKV- and DENV-infected brains that segregated from mock-infected samples (Fig. 4A). For ZIKV-infected brains, numerous genes were significantly induced compared to mock (Fig. 4B; Table 2). CXCL9 and CXCL10 were the most highly induced mRNA in the NanoString panel, followed by multiple interferon-related genes and components of the complement system (Table 2). Myl2 was the most significantly upregulated mRNA and the Rac/Rho small GTPase signalling components, Rac1 and RhoA were downregulated in the ZIKV-infected brain (Table 2). DENV responses were similar to ZIKV with numerous genes significantly induced in DENV-infected brains compared to mock (Fig. 4C; Table 2). Notably and in contrast to ZIKV, the highest upregulated mRNA during DENV-infection was for the complement alternative pathway activator, complement factor D (CfD; Table 2). A total of 23 mRNAs were different, and 56 were common in ZIKV compared to DENV-infected brain (Fig. 4C). Other complement components such as C3 were induced to comparable levels by DENV and ZIKV, but C2 and C4a were significantly upregulated by ZIKV but not DENV in the infected mouse brain. A comparable induction of C3 but a significantly greater induction of C2 in ZIKV compared to DENV-infected brain was confirmed by RT-PCR (Fig. 4D).

**Table 2:** Log-fold change of mRNA expression up- and downregulated in DENV- and ZIKV-infected brain. Adjusted *p*-value *q* < 0.05; > 1 log-fold change for upregulated genes

**Upregulated**						**Downregulated**		
**Gene**	**ZIKV**	**DENV**	**Gene**	**ZIKV**	**DENV**	**Gene**	**ZIKV**	**DENV**
**Cxcl9**	9.16	6.43	**Ifit2**	2.83		**Map3k5**	− 1.19	− 1.31
**Cxcl10**	8.11	5.41	**Itgb2**	2.82	2.51	**Il18**		− 1.14
**Irf7**	7.98	6.19	**Ccr1**	2.81	2.88	**Gnaq**		− 1.14
**Ifit1**	7.58	5.4	**C1ra**	2.74	2.35	**Plcb1**	-1	− 1.05
**Cfd**	7.37	7.28	**C4a**	2.63		**Map2k4**	-1	− 1.02
**Iigp1**	7.31	5.19	**Hspb1**	2.61	3.15	**Cfl1**		− 0.946
**Ifit3**	7.13	5.22	**Irf1**	2.59		**Mapk8**	− 0.922	− 1.03
**Oas1a**	7.05	4.97	**Ccl21a**	2.43	2.5	**Raf1**	− 0.901	-0.926
**Ifi27l2a**	6.91	5.41	**Stat2**	2.35		**Ptger3**	− 0.816	
**Ifi44**	6.89	5.01	**Arg1**	2.33	2.64	**Masp1**		− 0.8
**Mx2**	6.66	5.22	**Tlr9**	2.32		**Gnb1**	− 0.794	− 0.996
**Ccl5**	6.43	3.84	**Tgfb1**	2.31	2.63	**Tollip**	− 0.778	− 0.801
**Cfb**	6.42	4.92	**C1s**	2.28	2.57	**Grb2**	− 0.753	− 0.709
**Oas2**	6.29	4.26	**Nod1**	2.22	1.7	**Fxyd2**		− 0.736
**Oasl1**	6.14	4.45	**Tlr8**	2.17	1.84	**Prkcb**	− 0.694	− 0.635
**Ccl11**	6.06	6.42	**Cysltr1**	2.15	1.2	**Rps6ka5**	− 0.668	− 0.761
**C3**	6.04	5.87	**Tlr7**	2.08	1.56	**Map2k6**	− 0.658	− 0.591
**Il15**	5.67	5.08	**Irf5**	1.99	1.77	**Tcf4**	− 0.595	− 0.578
**Myl2**	5.59	6	**Tlr2**	1.97	1.31	**Bcl2l1**	− 0.502	− 0.654
**Alox12**	4.98	5.33	**Cebpb**	1.96	1.94	**Hif1a**	− 0.493	− 0.503
**Ptgir**	4.96	4.9	**C3ar1**	1.93	1.21	**Rac1**	− 0.386	− 0.247
**Stat1**	4.76	3.3	**Ltb**	1.89	1.38	**Rhoa**	− 0.329	
**Alox15**	4.45	4.91	**Ptgs2**	1.79	1.61	**Mapk1**	− 0.324	
**Hspb2**	4.38	4.48	**Pla2g4a**	1.71	1.51			
**H2-Eb1**	4.03	2.86	**Tyrobp**	1.59	1.48			
**Tnf**	3.86		**Cd86**	1.55	1.05			
**Tlr3**	3.78		**Relb**	1.54	1.47			
**Ccl2**	3.66	2.04	**Nod2**	1.5	1.06			
**Ccr2**	3.57	3.48	**Tgfb3**	1.46	1.67			
**H2-Ea-ps**	3.54	2.78	**Fos**	1.42	1.78			
**Mmp3**	3.36	3.69	**Mx1**	1.42				
**Ccl7**	3.16		**Ager**	1.4				
**Il1b**	2.88	2.31	**Maff**	1.31	1.32			
**C2**	2.88		**Tradd**	1.18				
**Ccl8**	2.86		**Tslp**	1.13	1.2			
**Il7**	2.85		**Shc1**	1.09	1.19			
**Hsh2d**	2.84		**Myd88**	1.02				
**Mmp9**	2.83	2.83	**Ltb4r1**	1				

**Fig. 5:**
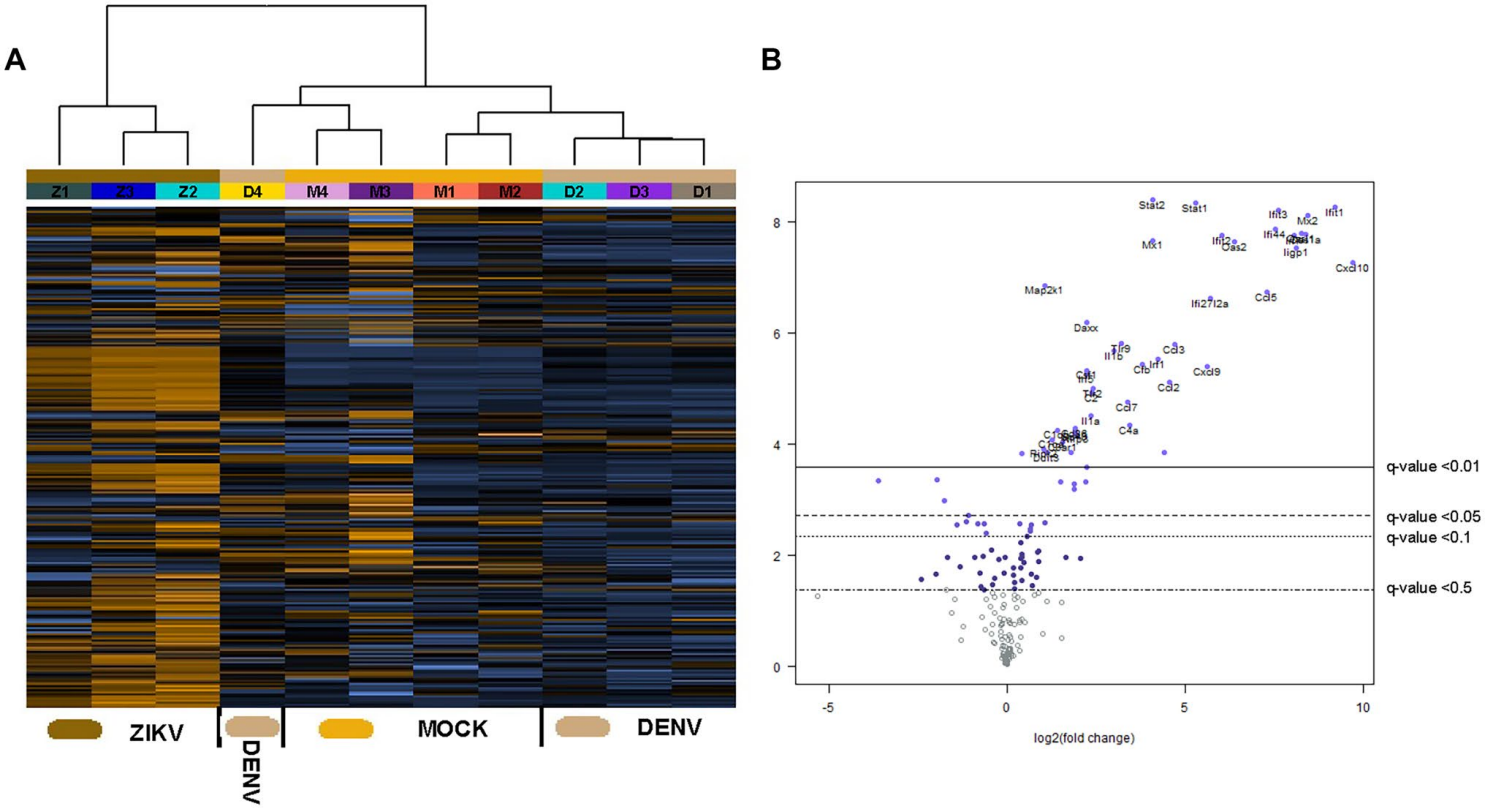
ZIKV induces inflammatory responses in the developing eye. Newborn Balb/c were mock (*n* = 4 eyes) or virally infected by injection of 5000 PFU of either DENV (*n* = 4 eyes) or ZIKV (*n* = 3 eyes), and total RNA extracted from the eye at six days pi, and subjected to NanoString nCounter analysis for a panel of mouse inflammatory-related genes. **A** Hierarchical clustering and heat map. **B** Volcano plots of mock vs ZIKV

Similarly, RNA from the eye at day 6 pi was subjected to NanoString analysis. Hierarchical clustering of eye responses demonstrate induction of mRNA changes in ZIKV-infected eyes, but, consistent with the variable detection of DENV RNA, DENV-infected samples did not segregate differently to mock-infected samples (Fig. 5A). Specific gene analysis, however, indicated 10 genes significantly increased in DENV-infected eyes and associated with an anti-viral and interferon-driven response (Table 3). For ZIKV-infected eyes, a number of genes were significantly induced compared to mock that reflected an antiviral and pro-inflammatory response (Fig. 5B; Table 3). CXCL10 was the most highly induced marker in the NanoString panel, and mRNA was induced for multiple components of the antiviral response (eg. MxA and Oas1), interferon related genes (eg. IFIT1, IRF7, IFI44), the complement system (complement factor B (CfB), C4a, and C2), and activated antigen presenting cells (Cd86 and Cd40). Interestingly, ZIKV-induced downregulated resistin-like molecule alpha (Retnla), while DENV-induced downregulation of arginase 1 (Arg1)—both factors which are involved in regulating Th2 responses (Table 3).

**Table 3:** Log-fold change of mRNA expression up- and downregulated in DENV- and ZIKV-infected eye. Adjusted *p*-value *q* < 0.05; > 1 log-fold change for upregulated genes

**Upregulated**						**Downregulated**		
**Gene**	**ZIKV**	**DENV**	**Gene**	**ZIKV**	**DENV**	**Gene**	**ZIKV**	**DENV**
**Cxcl10**	9.7		**Tlr9**	3.21		**Retnla**	− 3.6	
**Ifit1**	9.22	3.66	**Il1b**	3		**Arg1**		− 3.12
**Mx2**	8.46	2.72	**Tlr2**	2.41		**Alox5**	− 1.97	
**Oas1A**	8.38	3.23	**C2**	2.38		**Ccl24**	− 1.75	
**Oasl1**	8.27	2.42	**Il1a**	2.37		**Tbxa2r**	− 1.07	
**Iigp1**	8.12	2.34	**Csf1**	2.24				
**Irf7**	8.05	2.78	**Irf5**	2.24				
**Ifit3**	7.62	2.27	**Il6**	2.24				
**Ifi44**	7.54	2.91	**Daxx**	2.23				
**Ccl5**	7.31		**Il15**	2.21				
**Oas2**	6.4	2.38	**Nlrp3**	1.96				
**Ifit2**	6.03		**Cd86**	1.92				
**Ifi27l2a**	5.72	3.13	**Cd40**	1.92				
**Cxcl9**	5.61		**C1ra**	1.88				
**Stat1**	5.3	0.934	**Itgb2**	1.88				
**Ccl3**	4.72		**Tlr7**	1.8				
**Ccl2**	4.55		**C3ar1**	1.56				
**Tlr3**	4.41		**Nod1**	1.52				
**Irf1**	4.24		**C1qb**	1.41				
**Stat2**	4.09	0.56	**C1qa**	1.26				
**Mx1**	4.09		**Ddit3**	1.12				
**Cfb**	3.79		**Map2k1**	1.05				
**C4a**	3.44		**Ripk2**	1.03				
**Ccl7**	3.39		**Birc2**	0.431				

**Fig. 6:**
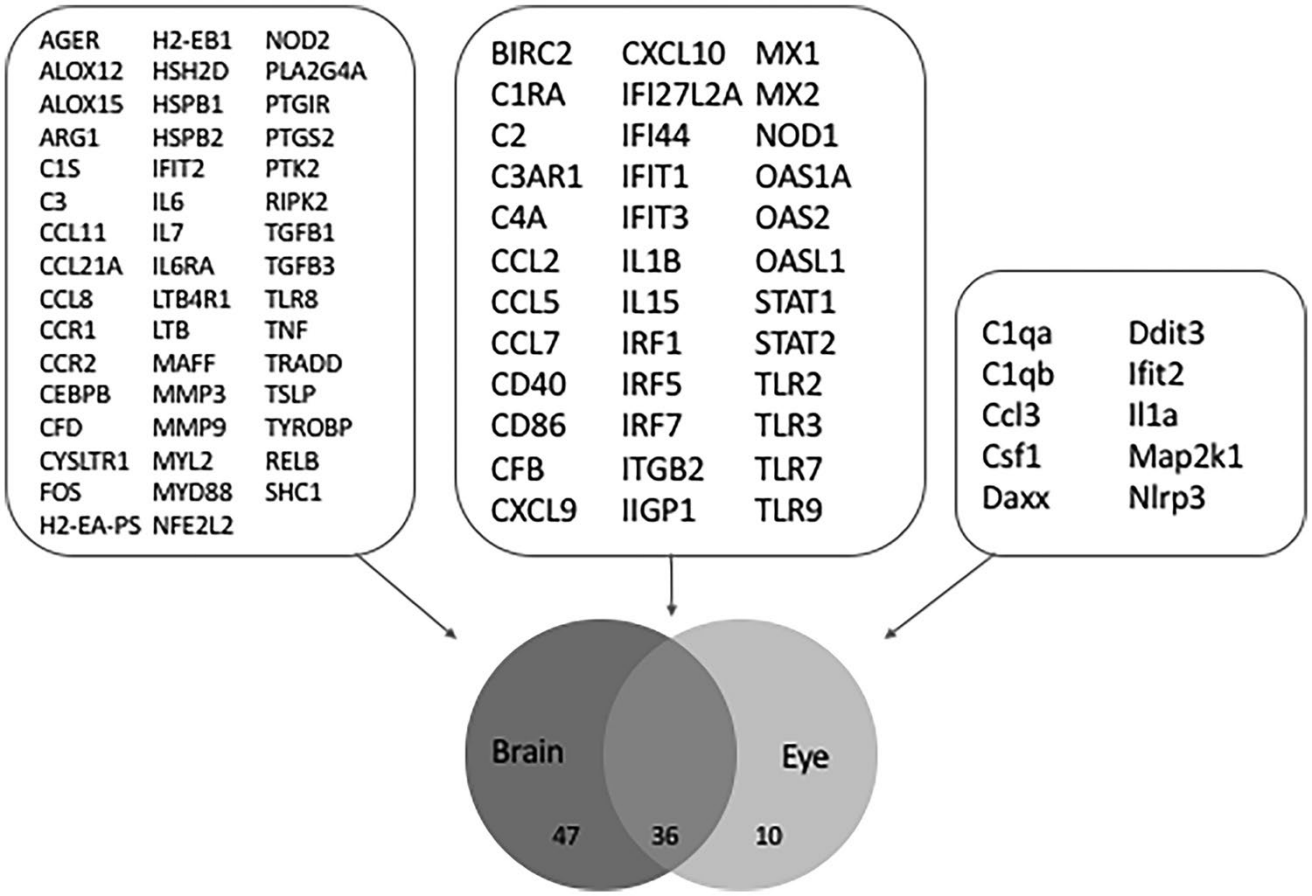
ZIKV induced both conserved and unique inflammatory responses in the developing eye and brain. RNA from newborn Balb/c mice that were mock- (*n* = 2 brain, *n* = 4 eyes) or ZIKV-infected was extracted and subjected to NanoString nCounter analysis for a panel of mouse inflammatory-related genes, as described in Figs. 4 and 5. mRNA for genes that were significantly upregulated by ZIKV-infection compared to mock in the eye and brain were segregated using a Venn diagram with the number of genes and the list of genes in unique and common quadrants shown

Since ZIKV consistently infected both the eye and brain, responses were compared in these tissues. Results demonstrate the induction of 36 mRNA’s common to both tissues including chemokines and components of the interferon, antiviral, and complement systems (Fig. 6). There was a unique induction of 47 mRNA’s in the brain, such as C3, CfD, and myosin light chain 2 (Myl2). Ten unique mRNA’s were induced in the eye but not the brain, such as colony stimulating factor 1 (CSF-1), DNA damage inducible transcript 3 (DdiT3), and NOD-, LRR-, and pyrin domain containing protein 3 (NLRP3) (Fig. 6; Table 4).

**Table 4:** Log-fold change of mRNA expression up- and downregulated in ZIKV-infected brain and eye. Adjusted *p*-value *q* < 0.05; > 1 log-fold change for upregulated genes

**ZIKV**								
**Upregulated**						**Downregulated**		
**Gene**	**Brain**	**Eye**	**Gene**	**Brain**	**Eye**	**Gene**	**Brain**	**Eye**
**Cxcl9**	9.16	5.61	**Stat2**	2.35	4.09	**Map3k5**	−1.19	
**Cxcl10**	8.11	9.7	**Arg1**	2.33		**Il18**		
**Irf7**	7.98	8.05	**Tlr9**	2.32	3.21	**Gnaq**		
**Ifit1**	7.58	9.22	**Tgfb1**	2.31		**Plcb1**	−1	
**Cfd**	7.37		**C1s**	2.28		**Map2k4**	−1	
**Iigp1**	7.31	8.12	**Nod1**	2.22	1.52	**Cfl1**		
**Ifit3**	7.13	7.62	**Tlr8**	2.17		**Mapk8**	−0.922	
**Oas1a**	7.05	8.38	**Cysltr1**	2.15		**Raf1**	−0.901	
**Ifi27l2a**	6.91	5.72	**Tlr7**	2.08	1.8	**Ptger3**	−0.816	
**Ifi44**	6.89	7.54	**Irf5**	1.99	2.24	**Masp1**		
**Mx2**	6.66	8.46	**Tlr2**	1.97	2.41	**Gnb1**	−0.794	
**Ccl5**	6.43	7.31	**Cebpb**	1.96		**Tollip**	−0.778	
**Cfb**	6.42	3.79	**C3ar1**	1.93		**Grb2**	−0.753	
**Oas2**	6.29	6.4	**Ltb**	1.89		**Fxyd2**		
**Oasl1**	6.14	8.27	**Ptgs2**	1.79		**Prkcb**	−0.694	
**Ccl11**	6.06		**Pla2g4a**	1.71		**Rps6ka5**	−0.668	
**C3**	6.04		**Tyrobp**	1.59		**Map2k6**	−0.658	
**Il15**	5.67	2.21	**Cd86**	1.55		**Tcf4**	−0.595	
**Myl2**	5.59		**Relb**	1.54		**Bcl2l1**	−0.502	
**Alox12**	4.98		**Nod2**	1.5		**Hif1a**	−0.493	
**Ptgir**	4.96		**Tgfb3**	1.46		**Rac1**	−0.386	
**Stat1**	4.76	5.3	**Fos**	1.42		**Rhoa**	−0.329	
**Alox15**	4.45		**Mx1**	1.42	4.09	**Mapk1**	−0.324	
**Hspb2**	4.38		**Ager**	1.4		**Retnla**		−3.6
**H2-Eb1**	4.03		**Maff**	1.31		**Arg1**		
**Tnf**	3.86		**Tradd**	1.18		**Alox5**		−1.97
**Tlr3**	3.78	4.41	**Tslp**	1.13		**Ccl24**		−1.75
**Ccl2**	3.66	4.55	**Shc1**	1.09		**Tbxa2r**		−1.07
**Ccr2**	3.57		**Myd88**	1.02				
**H2-Ea-ps**	3.54		**Ltb4r1**	1				
**Mmp3**	3.36		**Ccl3**		4.72			
**Ccl7**	3.16	3.39	**Il1a**		2.37			
**Il1b**	2.88	3	**Csf1**		2.24			
**C2**	2.88	2.38	**Il6**		2.24			
**Ccl8**	2.86		**Daxx**		2.23			
**Il7**	2.85		**Nlrp3**		1.96			
**Hsh2d**	2.84		**Cd86**		1.92			
**Mmp9**	2.83		**Cd40**		1.92			
**Ifit2**	2.83	6.03	**C3ar1**		1.56			
**Itgb2**	2.82	1.88	**C1qb**		1.41			
**Ccrl**	2.81		**C1qa**		1.26			
**C1ra**	2.74	1.88	**Ddit3**		1.12			
**C4a**	2.63	3.44	**Map2k1**		1.05			
**Hspb1**	2.61		**Ripk2**		1.03			
Irf1	2.59	4.24	**Birc2**		0.431			
Ccl21a	2.43							

**Fig. 7:**
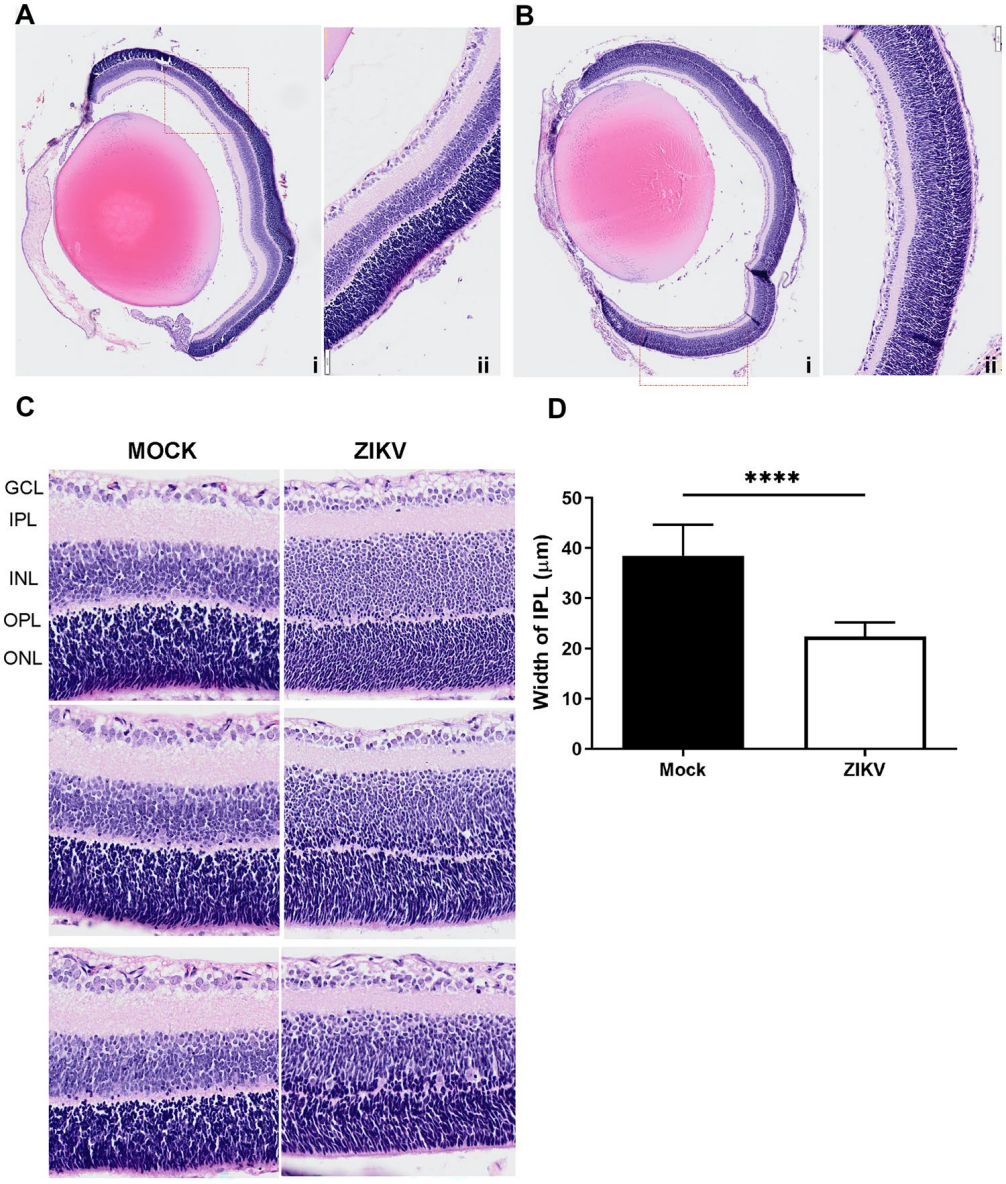
Morphological analysis of ZIKV-infection in the developing eye. Newborn Balb/c were mock- or ZIKV-infected at day 1 post-natal, and at day 6 pi eyes were harvested, fixed, sectioned, and stained with haematoxylin and eosin. **A** Mock (i) whole eye and (ii) × 40 magnification. **B** ZIKV-infected (i) whole eye and (ii) × 40 magnification. **C** × 100 magnification of retina from mock- and ZIKV-infected mice. Representative bright field images are shown. **D** Quantitation of retinal IPL layer. Data were analysed by Student’s *t*-test; * = *p* < 0.05. Images are representative of *n* = 4 (mock) and *n* = 5 (ZIKV) eyes

To investigate the morphological effect of ZIKV-infection in the eye, mock- and ZIKV-infected eyes taken at day 6 pi were fixed, sectioned. and stained with haematoxylin and eosin (Fig. 7). As expected, the retina of both mock- and ZIKV-infected eyes (7-day post-natal) was still immature and lacked pigmentation of the RPE layer (Fig. 7A). The structure of the retina, however, differed with a clear reduction in the thickness of the outer plexiform layer (OPL) in ZIKV-infected mice, accompanied by an apparent increase in the inner nuclear layer (INL), where the cells of the ZIKV-infected retina also appeared morphologically different (Fig. 7B, C). The boundary between the OPL and INL was difficult to define and thus were not quantitated, but quantitative measurement of the inner plexiform layer (IPL) demonstrated a significant reduction in width in the retinas from ZIKV-infected mice (Fig. 7D). No major cellular infiltrate was visualised in the ZIKV-infected eye (Fig. 7). Haematoxylin and eosin staining did not detect any gross morphological change in ZIKV-infected brain at day 6 pi (data is not shown).

## Discussion

DENV and other flaviviruses such as WNV and arboviruses such as CHIKV can infect the adult eye, with DENV associated with a number of different retinal pathologies (Merle et al. 2018; Oliver et al. 2019). DENV can cause infection in the adult brain, and although evidence for an effect of DENV on the central nervous system in patients is growing, DENV is generally not considered a neurotrophic flavivirus (Li et al. 2017). In contrast, ZIKV can cause major pathology in the brain and retinal defects following in utero infection of the developing fetus. Thus, definition of infection and host responses to both DENV and ZIKV in the eye and brain is of importance to help understand how these related viruses cause these different disease presentations. In this study, infection and host responses to ZIKV have been compared in vitro in adult retinal cell lines and in vivo in the developing brain and eye, alongside a laboratory strain of DENV.

It has been previously shown that DENV can infect RPE and exert functional changes (Carr et al. 2017). Additionally, DENV RNA and inflammatory responses can be detected in the mouse eye following either a systemic or intracranial infection (Norbury et al. 2020). Other studies have demonstrated that ZIKV can infect retinal cell types such as the retinal pigment epithelium (RPE), both primary RPE, the cell line ARPE-19, Muller cells and retinal endothelial cells in vitro (Singh et al. 2017; Zhao et al. 2017), and in a human fetal RPE cell line (Garcia et al. 2020) or iPSC-derived RPE (Simonin et al. 2019). The significance of this in particular is proposed to be due to the roles of the latter two cell types in forming a blood retinal barrier (BRB), where a breach of this barrier can allow the entry of ZIKV into the eye (Nelson et al. 2020; Roach and Alcendor 2017; Singh et al. 2017, 2018). In the study herein, ARPE-19 were most susceptible to both ZIKV and DENV, HREC became infected, but at low levels, and high levels of viral RNA with respect to the number of antigen positive cells were produced in Muller cells for both viruses. HREC demonstrated strong induction of type I IFN and inflammatory responses, consistent with our prior infection studies of DENV in human umbilical vein endothelial cells (Calvert et al. 2015) while Muller cells demonstrated a relatively poor type I IFN response. This confirms the literature and suggests that with our DENV and ZIKV strains, multiple cell types of the retina are susceptible to infection with the potential for a highly productive infection in Muller cells, as previously suggested to be a primary target in the retina (Zhao et al. 2017). Furthermore, all these cell types are potential contributors of their own unique inflammatory and antiviral responses that may be linked to restricting viral replication in the eye or promoting retinal inflammatory disease.

Using these same viral strains, infection in a 1-day old mouse demonstrated movement of DENV and ZIKV from systemic site of administration to the brain and eye. DENV infected the brain of all mice, but viral RNA was only detected unilaterally in the eye and not in all mice. The DENV strain used here is a laboratory clone of the New Guinea C DENV isolate, passaged in the mouse brain. This strain can infect the adult brain (Al-Shujairi et al. 2017) and eye following an intracranial challenge (Norbury et al. 2020) and replicate in eye cells in vitro (Carr et al. 2017), and as shown in vitro herein. Thus, this DENV strain has tropism for the eye but does not infect the developing mouse eye well. In contrast, ZIKV infected the brain and both eyes in all animals at day 3 pi with increased viral RNA at day 6 pi, at comparable levels in the brain and eye. From this, we suggest that DENV infection of the developing eye is restricted, either by the immune response or access to the eye itself. Consistent with this, ZIKV but not DENV infects the mouse eye in an intrauterine model of viral challenge (Shi et al. 2018).

ZIKV infection in the eye is supported by a number of studies in mouse models of systemic ZIKV infection in immunodeficient adult mice (Miner et al. 2016) or following direct inoculation into the eye (Singh et al. 2017; Zhao et al. 2017). These models result in high levels of ZIKV replication within choroidal endothelial cells and cells of the retina, identified as retinal pigment epithelial cells and Muller cells, consistent with in vitro studies (Singh et al. 2017; Zhao et al. 2017). ZIKV can induce an ocular pathology in the developing fetus following maternal infection (Mohr et al. 2018), and post-natally in a model of ZIKV infection during pregnancy in Rhesus macaques (Yiu et al. 2020). An alternative ZIKV developmental model is challenge of the 0–1 day post-natal (P0/1) mouse (Li et al. 2021), where mouse development from E15-P10 reflects third trimester gestation in humans (Chen et al. 2017; Workman et al. 2013). In this developing mouse model at P6-7, there is little replication as detected by direct staining of the eye or morphological change (Li et al. 2021). By P14-21 days of infection, there is significant morphological effect on brain and eye, with ZIKV causing apoptosis and destruction of the retinal ganglion layer, with progressive inflammation, loss of neurons, and vascular damage in the retina (Li et al. 2021). This is accompanied by neurological deficits and hind limb paralysis suggesting, similarly, a significant impact on the brain. The study here has analysed a similar model of infection at P0-1 and analysis at P6/7, where the developing pups are still healthy with no measurable clinical score or neurological defect. The retina is still immature, eyes are closed, the retinal epithelium is not yet pigmented, but there is detectable and increasing ZIKV-infection and a substantial inflammatory response. Importantly, this demonstrates responses and changes in the retina concurrent with responses in the brain and prior to major neurological deficits, suggesting the impact on the retina is not secondary to destruction of the brain. Although a prior study described little effect of ZIKV-infection on the retina at P6/7 (Li et al. 2021), in the study here, a clear retinal defect was observed with altered formation of OPL, INL and IPL layers, and morphological differences in the cells of the INL. Generation of the OPL has been staged in humans (Prameela Bharathan et al. 2021) and is important for development of the retina, where the OPL and IPL are regions for synaptic interactions of cells. Results here are thus consistent with an early defect in synaptic development in the retina of ZIKV-infected mice and would be expected to have significant impact on later vision outcomes. This has also been observed in the intrauterine ZIKV infection model, where by P7 the retina of ZIKV-infected mice is thinned, with loss of the OPL and starburst amacrine cells that are important for retinal circuitry (Shi et al. 2018).

Here, our study has also assessed ZIKV and DENV infection in the brain with results showing comparable replication for ZIKV and DENV and host responses detected with a NanoString inflammatory panel, segregating clearly from those of mock-infected mice. There were numerous conserved responses induced by ZIKV and DENV, with inflammatory and antiviral responses, as expected. Interestingly, CXCL9 and CXCL10 were the highest induced mRNA following ZIKV infection and was also induced but to a lesser degree by DENV. Since CXCL9 and CXCL10 have an important role in T-cell recruitment and favour a Th1 response, this suggests that ZIKV induces a stronger stimulus for a cellular recruitment to the brain. In contrast, CfD was the highest mRNA induced by DENV, suggesting a more local acting, complement driven response to infection. Both DENV and ZIKV induced a major upregulation of Myl2, a gene typically associated with cardiac growth, previously reported to be upregulated in ZIKV-infected myeloblasts (Riederer et al. 2022), and the relationship of this to non-muscle myosin and growth processes in the brain remains to be defined. Markers such as CD40, CD86, and CCL5 were increased, while induction of IFN-γ was not detected by NanoString, and CD4 and CD8 mRNA could not be detected by RT-PCR (data is not shown). This is suggestive of APC activation and monocyte responses. In contrast in the adult brain, infection with DENV induced CD8 + T-cell infiltration (Al-Shujairi et al. 2017), and the lack of T-cell responses here in the neonate is consistent with altered functions of neonatal T-cells (Rudd 2020). Interestingly, results show induction of components of the complement system that were common to both DENV and ZIKV in the brain such as CfB and C3, but C2 and C4a—components of the classical and lectin pathways, were only induced by ZIKV infection. The lack of induction of MASP1 and MASP2 suggests that the lectin pathway is not induced and that this increase in C2 and C4a reflects an increase in the classical pathway by ZIKV infection. Importantly, C1q, the starting substrate for the classical pathway, and C3b, the cleaved form of C3 following complement terminal pathway activation, both have roles in neuronal development and synaptic pruning in the brain (Warwick et al. 2021). C1q and C3 upregulation has also been described during ZIKV infection of the adult mouse brain and proposed to influence synapse formation (Figueiredo et al. 2019). Similarly, here, the increase in C1q and C3 combined with C2 and C4a following ZIKV infection may result in increased C1q cleavage and formation of excessive C3b and hence have a greater impact on neuronal development than DENV, which does not induce C2 and C4a and thus may maintain more C1q and less C3b. Only a few mRNA’s were downregulated, but notably, these included Rac1/RhoA, which are GTPases with known roles in maintaining a neural progenitor cell pool and neuronal development, that we have previously proposed may be relevant to ZIKV infection in the developing brain (Norbury et al. 2022).

Consistent with our observations of viral RNA in the eye, NanoString analysis demonstrated major responses to ZIKV but only induction of a small group of mRNAs for key antiviral factors in the eyes of DENV-infected mice. This supports our suggestion above, that DENV-infection induces an effective antiviral response that restricts infection in the eye. Notably, although the mRNA’s downregulated in the brain was altered by less than 1-log fold, in the eye, DENV and ZIKV induced an approximately 3-log fold decrease in Arg1 and Retnla, respectively. Both Arg1 and Retnla are influenced by Th2 cytokines and can regulate Th2 responses. Retnla-/-mice have increased Th2 responses, and conversely, Retnla overexpressing transgenic mice have lower Th2 responses (Lee et al. 2014; Nair et al. 2009). Arg1 is increased and associated with a Th2 environment (Bronte et al. 2003; Muraille et al. 2014). This suggests that the Th1/Th2 environment may be different with DENV downregulating Arg1 and thus decreased Th2 environment but ZIKV downregulating Retnla and thus promoting a Th2 environment. Consistent with a less inflammatory environment in the eye, ZIKV induced various C1 complement components in eye but did not induce key downstream activators of the AP such as CfD or the substrate for the terminal pathway, C3. In addition to these unique inflammatory profiles induced by ZIKV in the eye in comparison to the brain, ZIKV also induced CSF-1 and Ddit3, (also known as CHOP). CSF-1 is a growth factor responsible for supporting microglia in the brain (Elmore et al. 2014), which is produced by activated microglia and important in the retina for promoting photoreceptor survival (Jones and Ricardo 2013). Use of CSF1R blockers can deplete microglia and prevent microglial driven inflammatory damage in the eye (Kokona et al. 2018; Okunuki et al. 2019; Tang et al. 2020; Todd et al. 2019), and hence, it is unclear if an increase in CSF-1 would benefit the maintenance of photoreceptors or support a damaging inflammatory response to ZIKV infection driven by microglia, as we have observed, and can occur in vitro in Mueller cells. Ddit3 has been shown in the adult to contribute to retinal ganglion cell death (Wang et al. 2021) and consistent with the increase in Ddit3 seen here, and the pathology seen at later stages of ZIKV-infection in a similar model is loss of the RGC layer of the retina (Li et al. 2021; Shi et al. 2018). These changes in mRNA levels are seen in our study in the absence of major morphological change in the retina, such as RGC layer loss, and thus may be preceding triggers for this damage. Importantly, our study has observed a difference in the formation of the layers of the retina (INL and OPL) that form areas of synaptic interactions of retinal cells, which similar to the above discussions regarding the brain, could be driven by the complement system and the C1q and C3b roles in neurogenesis and synapse formation.

In conclusion, DENV and ZIKV both have capacity to infect cells of the adult eye in vitro, but ZIKV has a greater propensity to infect the developing mouse eye than DENV. These viruses induce distinct antiviral and inflammatory responses. ZIKV infects both the developing brain and eye at comparable levels and drives a retinal pathology and hosts responses including effects on factors, such as the complement system, that may influence brain and/or retinal development. Defining these responses and ways to dampen developmental impact without loss of control of viral replication may be of future benefit to lessen the burden of CZS and comparison to DENV, which infects the developing eye poorly, may help define responses that prevent infection of the developing eye.

## Supplementary Information

Below is the link to the electronic supplementary material.

Supplementary file1 (XLSX 88 KB)

Supplementary file2 (XLSX 95 KB)

## Data Availability

The authors confirm that the data supporting the findings of this study are available within the article and its supplementary materials.
